# Nucleolar Sequestration: Remodeling Nucleoli Into Amyloid Bodies

**DOI:** 10.3389/fgene.2019.01179

**Published:** 2019-11-21

**Authors:** Miling Wang, Michael Bokros, Phaedra Rebecca Theodoridis, Stephen Lee

**Affiliations:** ^1^Department of Biochemistry and Molecular Biology, Miller School of Medicine, University of Miami, Miami, FL, United States; ^2^Sylvester Comprehensive Cancer Center, Miller School of Medicine, University of Miami, Miami, FL, United States; ^3^Department of Urology, Miller School of Medicine, University of Miami, FL, United States

**Keywords:** heat shock (HS), acidosis, architectural RNA (arcRNA), Alzheimer’s disease, cellular dormancy, physiological amyloidogenesis, beta-amyloid protein

## Abstract

This year marks the 20th anniversary of the discovery that the nucleolus can temporarily immobilize proteins, a process known as nucleolar sequestration. This review reflects on the progress made to understand the physiological roles of nucleolar sequestration and the mechanisms involved in the immobilization of proteins. We discuss how protein immobilization can occur through a highly choreographed amyloidogenic program that converts the nucleolus into a large fibrous organelle with amyloid-like characteristics called the amyloid body (A-body). We propose a working model of A-body biogenesis that includes a role for low-complexity ribosomal intergenic spacer RNA (rIGSRNA) and a discrete peptide sequence, the amyloid-converting motif (ACM), found in many proteins that undergo immobilization. Amyloid bodies provide a unique model to study the multistep assembly of a membraneless compartment and may provide alternative insights into the pathological amyloidogenesis involved in neurological disorders.

## Nucleolar Sequestration: Visitors to the Nucleolus

The role of the nucleolus as the site of ribosome biosynthesis has been established since the mid-1960s (Perry, 1960; Perry, 1962; Miller and Beatty, 1969). Nucleoli are built around tandem repeats of ribosomal DNA (rDNA) in nucleolar organizing regions (NORs) and structurally dependent on active transcription of rDNA (Hernandez-Verdun, 2006; Raska et al., 2006). Each nucleolus consists of a tripartite organization which is classically defined by their different appearances under electron microscopy (EM) (Pederson, 2011): the fibrillar center (FC), where the RNA polymerase I machinery is active; the dense fibrillar component (DFC) that is enriched in fibrillarin (FIB1); and the granular component (GC) that harbors B23. RNA polymerase I activity is believed to occur at the interface between the FC and the DFC, while processing of newly synthesized ribosomal RNA (rRNA) and assembly with ribosomal proteins occur within the GC (Scheer and Hock, 1999; Thiry and Lafontaine, 2005). The traditional role of the nucleolus as a hub of rRNA synthesis and ribosome assembly has been the subject of many excellent literature surveys (Boisvert, 2007; Pederson, 2011; Nemeth and Grummt, 2018). This review focuses on a lesser known phenomenon originally coined “nucleolar sequestration,” which describes the ability of the nucleolus to sequester regulatory proteins in response to cellular cues (Emmott and Hiscox, 2009; Pederson and Tsai, 2009; Boulon, 2010).

2019 marks 20 years since nucleolar sequestration was first hypothesized (Bachant and Elledge, 1999) following the discoveries that cell cycle regulator Cdc14 phosphatase and E3 ubiquitin ligase MDM2 could be temporarily localized in nucleoli to affect cell cycle progression (Shou et al., 1999; Visintin et al., 1999; Weber, 1999; Lohrum, 2000; Bernardi et al., 2004). In *Saccharomyces cerevisiae*, Cdc14 is sequestered in the nucleolus by Cfi1/Net1 until anaphase onset when it is released to promote exit from mitosis (Shou et al., 1999; Visintin et al., 1999). In mammalian cells, nucleolar sequestration of MDM2 prevents it from binding and exporting p53 into the cytoplasm for degradation (Weber, 1999; Lohrum, 2000; Bernardi et al., 2004). This stabilizes p53 in the nucleus, where it acts as a transcription factor that promotes growth arrest or apoptosis. The E3 ubiquitin ligase von Hippel–Lindau protein (VHL) is another example of nucleolar sequestration (Mekhail, 2004). VHL promotes the degradation of the transcription factor hypoxia-inducible factor (HIF) under normal oxygen conditions. Low extracellular pH triggers nucleolar sequestration of VHL, enabling HIF to evade degradation and promote transcription of target genes involved in oxygen homeostasis. **Table 1** summarizes the reports of nucleolar sequestration that have been observed for many other proteins under various cellular conditions. Taken together, these underscore a fundamental cellular strategy that uses the nucleolus to regulate protein dynamics in response to cellular cues.

**Table 1:** List of the proteins whose activities have been reported to be regulated by nucleolar sequestration.

Protein symbol	Full name	Stimulus	Nucleolar response	Model system	Reference
Cdc14	Cell division cycle 14	Anaphase	Release	*S. cerevisiae*	(Shou, 1999) (Visintin et al., 1999)
Pch2	Pachytene checkpoint 2	Meiotic prophase arrest	Release	*S. cerevisiae*	(San-Segundo and Roeder, 1999)
MDM2	Murine double minute 2 homolog	Ribosomal stress DNA damage	Capture	Mammalian	(Weber, 1999) (Lohrum, 2000) (Bernardi, 2004)
hTERT	Human telomerase reverse transcriptase	Transformation, DNA damage Ionizing radiation	Release Capture	Mammalian	(Wong et al., 2002)
c-Myc	Proto-oncogene c-Myc	Proteasomal stress	Capture	Mammalian	(Arabi, 2003)
ADAR2	Adenosine deaminase that acts on RNA 2	Inhibition of rRNA synthesis	Release	Mammalian	(Sansam et al., 2003)
VHL	von Hippel Lindau tumor suppressor	Extracellular acidosis	Capture	Mammalian	(Mekhail, 2004)
RelA	p65 subunit of transcription factor NF-κB	Aspirin, serum withdrawal, UV-C radiation	Capture	Mammalian	(Stark and Dunlop, 2005) (Chen and Stark, 2017)
Polycomb	Polycomb	Cell differentiation	Capture	*D. melanogaster*	(Chen, 2005)
Hand1	Heart and neural crest derivatives expressed 1	Cell differentiation	Capture/Release	Mammalian	(Martindill et al., 2007)
Hsc70	Heat shock chaperone 70	Recovery from heat shock	Capture	Mammalian	(Banski, 2010)
Ulp1	Small ubiquitin-related modifier (SUMO) protease	Alcohol	Capture	*S. cerevisiae*	(Sydorskyy et al., 2010)
p53	Cell cycle regulator; tumor suppressor	Proteasomal inhibition (MG132)	Capture	Mammalian	(Kruger and Scheer, 2010) (Latonen, 2011)
Piwi	piRNA binding protein	Heat shock	Capture	*D. melanogaster*	(Mikhaleva, 2018)

## Nucleolar Sequestration: A Case of Protein Immobilization

### The Nucleolar Detention Centers

Pioneering work by Phair and Misteli (2000) showed that proteins are highly mobile and rapidly exchange between affinity interactions and the cellular milieu. The nucleolus is considered a dynamic droplet, assembled by demixing of its three sub-structural liquid phases (i.e., FC, DFC, and GC), which are composed of highly mobile proteins (Brangwynne et al., 2011; Feric et al., 2016; Hult et al., 2017; Sawyer et al., 2019). It is remarkable, then, that proteins undergoing nucleolar sequestration are non-dynamic or immobile (Mekhail, 2004; Mekhail, 2005; Audas et al., 2012; Jacob, 2013). This has been demonstrated for Cdc14 (Tomson et al., 2009), MDM2 (Audas et al., 2012), VHL (Mekhail, 2005), RNF8 (Mekhail, 2007), DNMT1 (Audas et al., 2012), and Piwi (Mikhaleva, 2018), amongst other proteins. Even resident nucleolar proteins such as RNA polymerase I subunit RPA16, Pescadillo, and SENP3 can undergo cycles of mobility/immobility (Jacob, 2013). For example, VHL switches from a highly dynamic, uniform distribution under standard growth conditions (21% O_2_, pH 7.4) to an immobilized state in the nucleolus on exposure to extracellular acidosis (1% O_2_, pH 6.0) (Mekhail, 2005). Only upon neutralization of extracellular pH is VHL released from the nucleolus to return to its original distribution and mobility (Mekhail, 2005). These nuclear foci that contain sequestered/immobile proteins were originally called “nucleolar detention centers,” as targets are both localized and detained within the nucleolus, unable to freely diffuse elsewhere (Mekhail, 2005; Audas et al., 2012b; Jacob, 2013). From this perspective, the function of nucleolar detention or immobilization is to temporarily inactivate relevant proteins, inhibiting their access to downstream effectors. Just as possible, though, is that the clustering of detained proteins may render an enzymatic reaction more efficient. For example, immobilized nucleolar Cdc14 maintains Spo12 dephosphorylation to regulate cell cycle progression (Tomson et al., 2009). In addition, Piwi switches from its canonical role as a non-nucleolar transposable element repressor to a rDNA-specific repressor when it is sequestered in the nucleolus (Mikhaleva, 2018). From these studies, it is clear that cells have evolved a strategy to regulate molecular networks by reversibly switching proteins between a mobile and an immobile state. Whether nucleolar sequestration represents a loss- or gain-of-function might depend on the proteins undergoing immobilization.

### Mechanisms of Nucleolar Sequestration

The ability of the nucleolus to sequester a wide variety of proteins in various cellular contexts suggests multiple mechanisms for nucleolar sequestration. Pathway analysis of interactions between resident nucleolar proteins and visitor proteins indicates that sequestered proteins interact either directly or indirectly, with the same small subset of “hub” nucleolar proteins, which primarily includes B23/NPM and Nucleolin (Emmott and Hiscox, 2009). Nucleolar retention of highly dynamic proteins through interactions with less mobile “hub” nucleolar partner(s) anchored by multivalent protein and RNA interactions contribute to nucleolar plasticity (Mitrea et al., 2016). Cdc14 is anchored in the nucleolus for most of the cell cycle through its association with Cfi1/Net1 (Shou et al., 1999; Visintin et al., 1999). During anaphase onset, a signaling cascade of phosphorylation inhibits the interaction of Cdc14 with Net1, releasing it to act as a mitotic exit activator (Azzam et al., 2004). ARF binding to MDM2 unmasks a cryptic nucleolar localization signal (NoLS) within its C-terminal RING domain, which is essential for MDM2 nucleolar sequestration (Tao and Levine, 1999; Weber, 1999; Lohrum, 2000; Weber et al., 2000). During DNA damage and acidosis, MDM2 is shuttled into the nucleolus by direct binding to PML (promyelocytic leukemia protein) (Bernardi et al., 2004), itself a target of nucleolar sequestration (Mattsson, 2001). MDM2 can also be sequestered in nucleoli by binding to ATP, independently of ARF (Poyurovsky et al., 2003). Alternatively, MDM2 binds ribosomal proteins (e.g., RPL5 and RPL11) that are released into the nucleoplasm during ribosomal stress, which stabilizes p53 (Boulon, 2010; Liu et al., 2016). Mapping analysis of VHL identified an approximately 30-amino acid fragment referred to as a nucleolar detention signal (NoDS) that is necessary and sufficient to immobilize proteins in nucleoli (Mekhail, 2007). The NoDS is composed of an arginine/histidine-rich sequence followed by two or more hydrophobic LXV motifs where X can be any hydrophobic residue (e.g., LWL, LLV, LFV, and LQV). A survey of proteins containing NoDS identified many candidates harboring this motif, including DNA methyltransferase I DNMT1, PML, and DNA polymerase delta catalytic subunit POLD1, all shown to be immobilized in nucleoli under extracellular acidosis (Mekhail, 2007; Audas et al., 2012a). The NoDS interacts with inducible long noncoding RNA derived from the ribosomal intergenic spacer (rIGSRNA) (Audas et al., 2012a). Silencing of rIGSRNA is sufficient to prevent both the formation of nucleolar detention centers and immobilization of NoDS-containing proteins (Audas et al., 2012a). The identification of the NoDS in many proteins provided evidence that nucleolar sequestration is a common cellular strategy to regulate protein function (Mekhail, 2007).

## Biochemical Properties of Nucleolar Detention Centers

### Nucleolar Detention Centers Display Amyloidogenic-Like Characteristics

**Figure 1:**
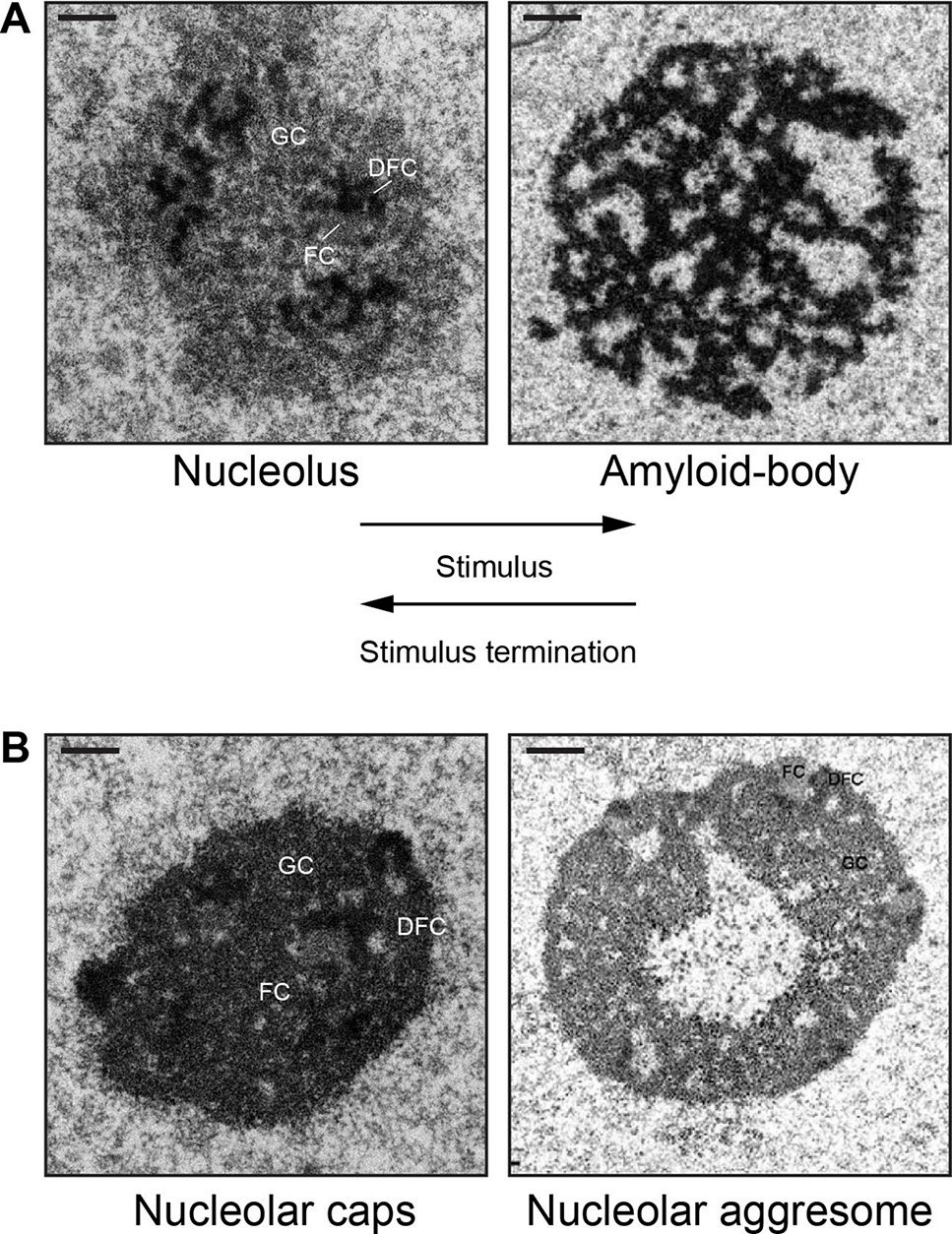
Nucleolar sequestration: the reversible remodeling of the nucleolus into an amyloid body. **(A)** During stimuli (heat shock or extracellular acidosis), the tripartite nucleolus undergoes a dramatic transformation into electron-dense fibrillar organization that characterizes an amyloid body. The fibers contain immobilized proteins in an amyloid-like state. After stimuli termination, an amyloid body is disaggregated and transforms back into the tripartite nucleolus. **(B)** The fibrillar amyloid bodies are distinct from the amorphous, electron-dense nucleolar caps (16 h cisplatin) or the electron-light nucleolar aggresomes (16 h MG132). *FC,* fibrillar component; *DFC*, dense fibrillar component; *GC*, granular component. Scale represents 1 µm. Amyloid body and nucleolar aggresome taken from (Audas, 2016) and (Kruger and Scheer, 2010), with permission.

In various physiological settings, proteins with limited mobility often display amyloid-like properties (Kayatekin et al., 2014; Berchowitz et al., 2015). Amyloidogenesis is the process whereby soluble proteins assemble into aggregates known as amyloid fibrils (Knowles et al., 2014). Because of their association to neurodegenerative disorders, including Alzheimer’s and Parkinson’s disease, amyloids were classically perceived as exclusively toxic protein aggregates (Schnabel, 2010; Knowles et al., 2014). The discovery of functional amyloid Pmel17 in 2006 challenged the notion that the amyloid state is merely pathological (Fowler, 2006). Since then, several groups have reported the existence of functional/physiological amyloids in different organisms (Chiti and Dobson, 2006; Fowler, 2007; Maji et al., 2009; Falabella et al., 2012; Fowler and Kelly, 2012; Li et al., 2012; Kayatekin et al., 2014; Berchowitz et al., 2015; Tang et al., 2015; Saad, 2017; Cereghetti, 2018). Nucleolar detention centers composed of proteins such as VHL, MDM2, POLD1, etc., share many properties beyond immobility that are associated with the amyloid state. First, these nuclear foci stained positive with amyloidophilic dyes such as Congo red, Thioflavin S/T, and Amylo-Glo, all of which recognize different biochemical features of amyloids (Audas et al., 2016). Second, the nucleolar detention centers stain positive for the OC fibril antibody that specifically targets the amyloid fibril conformation (Kayed et al., 2007). Third, immobilized proteins found in these nuclear foci displayed biochemical properties associated with amyloids, including resistance to proteinase K, insolubility in common detergents, and can only be dissociated into monomers by SDS/high temperature (Audas et al., 2016). Arguably the utmost unique feature of amyloid bodies is their electron-dense fibrillar organization, which would be as predicted for a condensate enriched in amylogenic proteins (Jacob, 2013; Audas et al., 2016). Consequently, the terms “nucleolar detention centers” and NoDS were replaced with “amyloid bodies” (A-bodies) and “amyloid-converting motif” (ACM), respectively, to reflect the transformation of the nucleolus into an A-body, a molecular prison of proteins in their amyloid-like state. **Figure 1A** shows an example of this dramatic and reversible transformation of the tripartite nucleolus into the fibrillar A-body in cells responding to stimuli.

### Amyloid Bodies Are Distinct From Liquid-Like Biomolecular Condensates and Other Nucleolar Organizations

Purification and mass spectrometry analysis have identified hundreds of cellular proteins that are captured in A-bodies, many of which were shown to undergo immobilization by photobleaching experiments (Mekhail, 2007; Audas et al., 2012a; Audas et al., 2016). Because A-bodies contain an array of immobilized proteins in an amyloid-like state, we suggested the term “systemic physiological amyloidogenesis” to describe A-body biogenesis, in keeping with the original terminology to describe functional amyloids (Chiti and Dobson, 2006; Fowler, 2007; Maji et al., 2009). Other laboratories have also proposed that A-body formation represents an amyloidogenic liquid-to-solid phase transition (Lyons and Anderson, 2016; Latonen, 2019; Hall et al., 2019) and used the terms “solid,” “solid-like,” or “non-dynamic” biomolecular condensates to describe this membraneless organelle (Lyons and Anderson, 2016; Weber, 2017; Cereghetti, 2018; Holehouse and Pappu, 2018; Woodruff et al., 2018; Itakura et al., 2018; Fay and Anderson, 2018; Latonen, 2019). Prior to the discovery of A-bodies, the terms “liquid-to-solid phase transition” and “solid” had been reserved to describe the formation of pathological aggregates (Patel et al., 2015; Murakami et al., 2015; Jain and Vale, 2017; Mateju et al., 2017; Peskett, 2018; Posey et al., 2018). Other physiological examples of “solid-like” structures include Balbiani bodies observed in *Xenopus* (Boke et al, 2016) and pH-regulated fluid-to-solid transition of the cytoplasm in yeast (Munder et al, 2016). The fibrous, amyloid-like characteristic of the A-body differentiates it from other biomolecular condensates that display liquid-like properties (Brangwynne et al., 2009; Brangwynne et al., 2011; Weber and Brangwynne, 2012; Zhu and Brangwynne, 2015; Banani, 2017; Shin and Brangwynne, 2017), such as stress granules, nucleoli, and paraspeckles, amongst others (Hodges, 1998; Gall, 2000; Lamond and Spector, 2003; Rizzi et al., 2004; Dellaire and Bazett-Jones, 2004; Valgardsdottir, 2005; Parker and Sheth, 2007; Sasaki and Hirose, 2009; Bond and Fox, 2009; Machyna et al., 2013; Pederson, 2011; Protter and Parker, 2016). Liquid-like biomolecular condensates are dynamic, their constituents are mobile, they do not form fibers detectable by EM nor do they typically stain with amyloidophilic dyes (Phair and Misteli, 2000; Shin and Brangwynne, 2017; Weber, 2017). The biochemical and biophysical differences between dynamic, liquid-like, and non-dynamic or solid-like condensates are summarized within **Table 2**.

**Table 2:** Biochemical, biophysical, and dynamic properties of liquid-like condensates or solid-like condensates with amyloid characteristics.

	Liquid-like condensates	Solid-like condensates
Examples	Cytoplasm stress granules (Protter and Parker, 2016) P-bodies (Parker and Sheth, 2007) Nuclear stress granules (Rizzi et al., 2004; Valgardsdottir et al., 2005) Cajal bodies (Gall, 2000; Machyna et al., 2013) Nuclear speckles (Lamond and Spector, 2003) Nuclear paraspeckles (Bond and Fox, 2009; Sasaki and Hirose, 2009) Nucleoli (Pederson, 2011) PML nuclear bodies (Hodges et al., 1998; Dellaire and Bazett-Jones, 2004)	Amyloid bodies (Audas, 2016) Balbiani bodies (Boke, 2016)
Protein mobility	Proteins are mobile; continuously exchanging with the structure and the surrounding milieu	Proteins are immobile; engaged in strong intermolecular interactions
Shape	Spherical	Spherical or fibrous
Biochemical and biophysical characteristics	• Structure is dynamic; exhibiting properties of water droplets:Fluid • Cycles of fusion (coalescence) and fission • Wetting behavior • Flows under shear force	• Structure is non-dynamic; exhibiting properties of amyloids:Static • Fibrillar organization • Positive staining with amyloidophilic dyes (e.g., Congo red) • Resistant to proteinase K • Insoluble in common detergents • Cross-β diffraction pattern
Material properties	Viscous	Elastic
Function	Biochemical reactions	Cell dormancy
Mechanism	Liquid–liquid phase separation	Liquid-to-solid phase transition

A-bodies may also be contrasted from other stress-induced nucleolar structures, namely, nucleolar caps (Shav-Tal et al., 2005) and nucleolar aggresomes (Latonen, 2011; Latonen, 2011; Latonen, 2019) (**Figure 1B**). Transcriptional inhibition of Pol I results in nucleolar segregation, in which the FC and GC phases separate, perhaps as a consequence of changes in surface tension (Shin and Brangwynne, 2017) resulting in the formation of perinucleolar caps that surround the segregated nucleolus (Shav-Tal et al., 2005). Proteasomal inhibition induces nucleolar inclusions called nucleolar aggresomes that contain proteins marked for degradation, and various RNAs (Latonen, 2011). By EM, A-bodies have a unique fibrillar organization characteristic of amyloids, whereas nucleolar caps appear as electron-dense amorphous structures and nucleolar aggresomes are cavernous, occupying a large electron-light central space of the nucleolus (Kruger and Scheer, 2010) (**Figure 1B**). Interestingly, while the formation of nucleolar caps and A-bodies is accompanied by a redistribution of nucleolar components and subsequent arrest of ribosomal biogenesis (Shav-Tal et al., 2005; Mekhail, 2006; Jacob, 2013), nucleolar aggresomes require transcriptionally active nucleoli to form (Latonen, 2011; Latonen, 2011), with all nucleolar components intact and visible by EM. Additionally, proteins are dynamically sorted into nucleolar caps and remain mobile (Shav-Tal et al., 2005), while several proteins in A-bodies and nucleolar aggresomes are immobile (Mekhail, 2005; Audas et al., 2012a; Jacob, 2013; Audas et al., 2016). Another recently identified nucleolar stress body, nucleolar amyloid bodies (NoABs), is induced by prematurely terminated peptides that diffuse through the nuclear pores and aggregate within the nucleolus (Mediani et al., 2019). EM has not been reported for NoABs. It remains to be tested if NoABs are rIGSRNA-dependent, as are A-bodies, and if prematurely terminated peptides are involved in nucleolar sequestration of full-length proteins. While there are definitive similarities between the different stress-induced nucleolar organizations and that proteins can undergo sequestration in nucleolar aggresomes (both are discussed in (Latonen, 2019), A-bodies represent a unique nucleolar structure based on their fibrous properties and dependency on rIGSRNA.

## Protein Immobilization Into A-Bodies: A Choreographed Multistep Pathway

How membraneless subcellular condensates maintain their unique identities and how proteins and/or RNA are sorted into these condensates remain subjects of active research. New insights into the biophysical properties of biomolecular condensates have demonstrated unexpected links between the sequence-encoded information in protein and RNA components of compartments and the material properties they impart (Kato et al., 2012; Altmeyer et al., 2015; Brangwynne et al., 2015; Elbaum-Garfinkle et al., 2015; Molliex et al., 2015; Nott et al., 2015; Pak et al., 2016; Shin and Brangwynne, 2017; Weber, 2017; Langdon, 2018). Recent studies demonstrated the importance of RNA-binding proteins with prion-like, low-complexity domains in forming biomolecular condensates. Equally as exciting has been the work done to uncover the specific sequences or structural elements embedded in architectural RNA that dictate the biophysical properties of biomolecular condensates (Chujo, 2017; Chujo and Hirose, 2017; Yamazaki et al., 2018; Hirose et al., 2019). With these new tools, we have begun to understand how specific elements within RNA and proteins are involved in condensate biogenesis.

### A Working Model of A-Body Biogenesis

**Figure 2:**
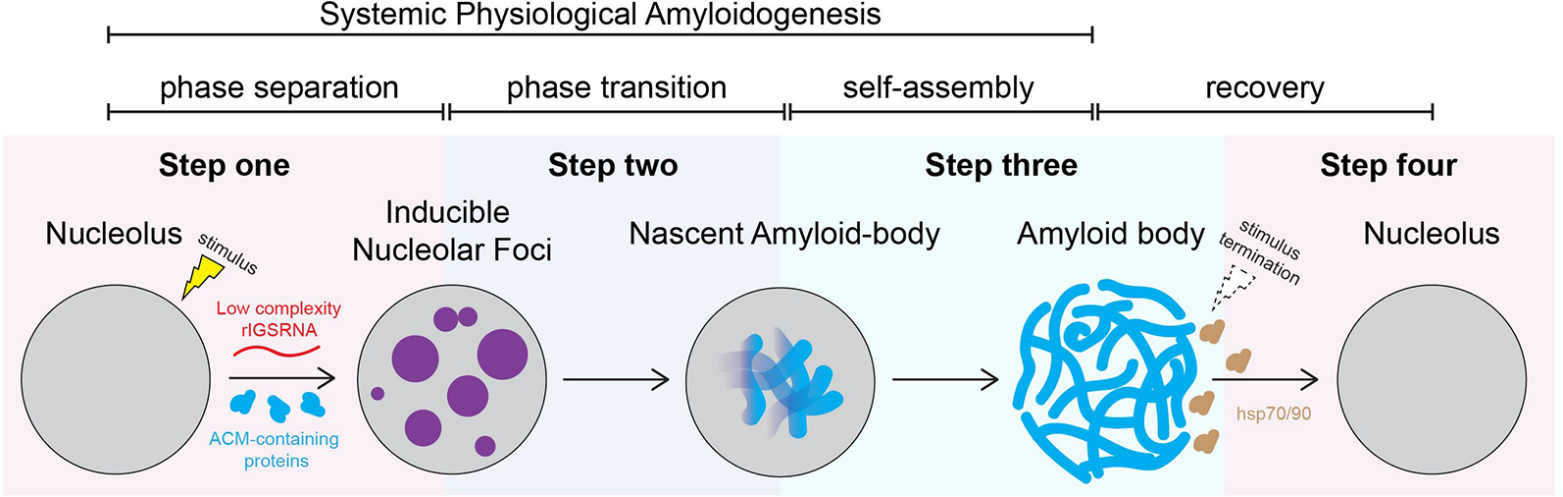
Working model: amyloid body biogenesis is a precisely choreographed routine. We propose that, on stimulus, low-complexity ribosomal intergenic spacer RNA (rIGSRNA) derived from the rDNA intergenic spacer accumulate in the nucleolus. Step 1: Low-complexity rIGSRNA interact with short cationic peptides, such as the R/H-rich sequence of the ACM (formally NoDS), to form nucleolar liquid-like foci. Step 2: Local concentration of proteins with amyloidogenic propensity in the foci triggers physiological amyloidogenesis and generates nascent amyloid bodies (A-bodies). Step 3: Once seeded, nascent A-bodies self-assemble into fibrillar, solid-like A-bodies. A-bodies enable cells to rapidly and reversibly store a large array of proteins and enter cellular dormancy in response to stress. Step 4: Upon recovery/stimulus termination, A-body disaggregation is mediated by heat shock protein (hsp) chaperones 70 and 90. Through these steps, A-body biogenesis may represent a physiological liquid-to-solid phase transition.

**Figure 2** proposes a stepwise working model of A-body biogenesis that integrates different opinions in the literature, showcasing this process as a precisely choreographed multistep routine rather than simply a random aggregation of misfolded proteins. The first step in the formation of A-bodies is the appearance of several transient foci that are distinct from known nucleolar layers (Wang et al., 2018). These foci are spherical, contain mobile proteins, and can undergo fusion, thereby displaying some of the properties associated with dynamic condensates. An interesting feature of the inducible nucleolar foci is that they stain positive for 1-anilino-8-naphthalenesulfonate (ANS), a dye specific for hydrophobic regions of proteins and used *in vitro* to detect the molten globule state, a precursor of amyloid fibrils (Booth, 1997). EM revealed that ANS-positive foci correspond to electron-dense, amorphous aggregates concomitant with loss of the typical tripartite organization of transcriptionally active nucleoli. In the second step, the stimulus-induced foci rapidly mature into Congo red-positive aggregates that contain immobilized proteins, limiting their ability to diffuse within the nucleolus. We coined these maturing foci “nascent A-bodies.” Photobleaching analysis showed that, once formed, nascent A-bodies expand by directly capturing and immobilizing free proteins (step 3) (Wang et al, 2018). Maturation of A-bodies terminates once the pools of cellular mobile proteins have been depleted, culminating in a distinct fibrous organization. Disassembly of A-bodies occurs within 1–2 h after stimulus termination, a process that requires heat shock proteins hsp70 and hsp90 (step 4) (Audas et al., 2016).

### Low-Complexity rIGSRNA Drive Formation of Inducible Nucleolar Foci

**Figure 3:**
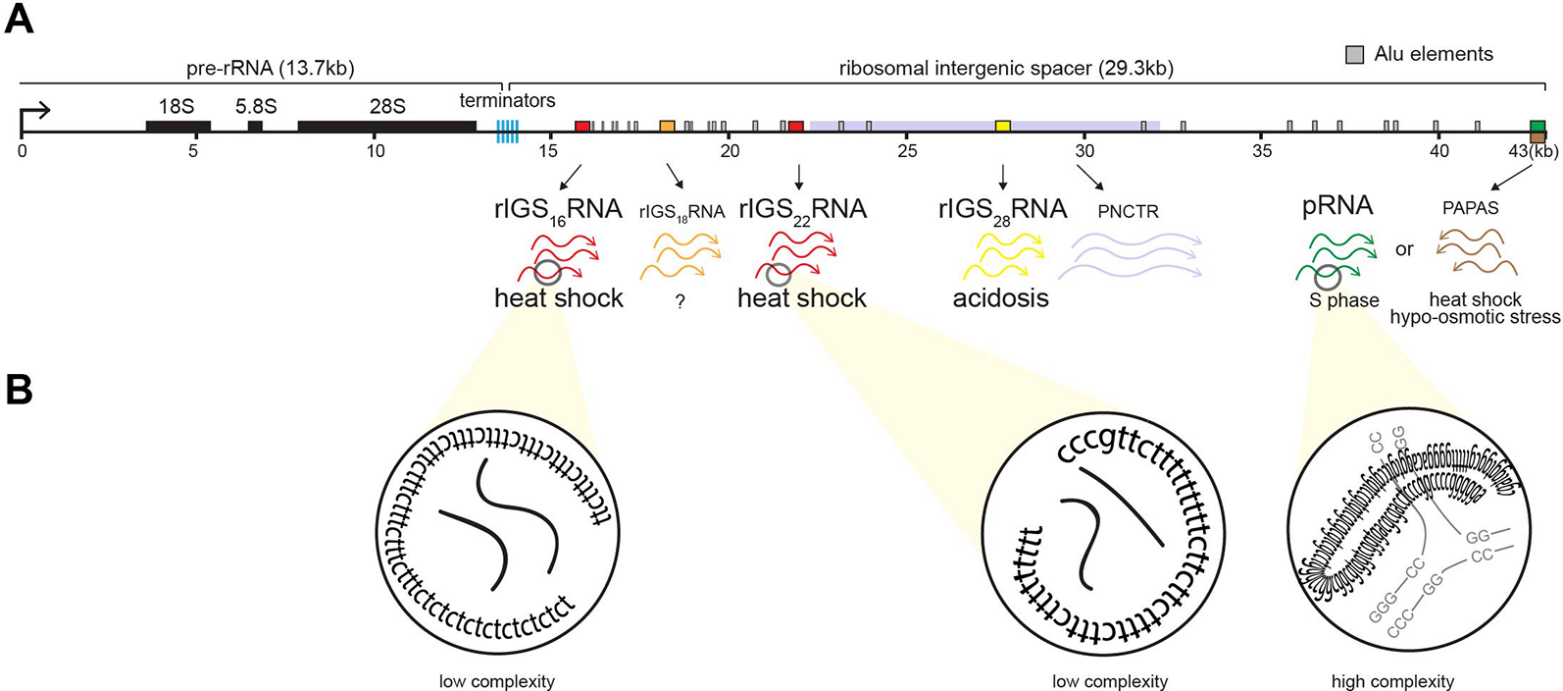
Induction of non-coding RNA (ncRNA) from the ribosomal cassette. **(A)** Schematic of a single human rDNA repeat unit, which is composed of a ∼13-kb pre-rRNA coding region flanked by a ∼30-kb intergenic spacer (rIGS). The rIGS transcribes several functional non-coding RNA. Stimuli-specific loci of rIGS produce ribosomal intergenic spacer RNA (rIGSRNA) required for A-body formation. rIGS_28_RNA and rIGS_16_RNA/rIGS_22_RNA are produced under acidotic (*yellow*) and heat shock (*red*) conditions, respectively. No function has been ascribed to rIGS_18_RNA yet. Other ncRNA found in the rIGS include a >10-kb transcript called PNCTR (pyrimidine-rich non-coding transcript) involved in PTBP1 binding (purple), *pRNA* (green), and antisense PAPAS (brown) involved in rRNA regulation, as well as Alu element-derived (gray boxes) RNA involved in nucleolar architecture. **(B)** rIGSRNA contain low-complexity sequences comprising of long dinucleotide repeats, as determined by RNA sequencing, RT cloning, and RNA-FISH. This is in contrast to other ncRNA that display high complexity, i.e., possess secondary structure, such as pRNA.

In mammals, the nucleolus is organized around a scaffold of ∼400 rDNA tandem repeats of 43 kb, of which approximately half are transcriptionally active (Nemeth and Grummt, 2018; Sharifi and Bierhoff, 2018). Each repeat consists of an rDNA enhancer/promoter located directly upstream of rRNA genes separated by a ribosomal intergenic spacer (rIGS) of variable length and organization (Gonzalez and Sylvester, 1995; Smirnov, 2016) (**Figure 3A**). The rIGS is an enigmatic region of the human genome historically, and erroneously, called the “non-transcribed region” (Smirnov, 2016). Interestingly, in recent years, species conservation (Agrawal and Ganley, 2018) and functional studies have demonstrated that these regions of the genome are transcriptionally active, generating various non-coding RNA (ncRNA) (Jacob, 2012; Audas and Lee, 2016). These ncRNA from the rIGS are involved in regulating rRNA expression (Mayer, 2006; Mayer et al., 2008; Schmitz, 2010; Zhao, 2016; Zhao, 2016; Zhao, 2018) and thereby responsible for maintaining a significant fraction of the rDNA cassettes in a heterochromatic, transcriptionally silent chromatin structure (Grummt and Pikaard, 2003; Santoro, 2005), controlling PTBP1-regulated alternative splicing (Yap, 2018), and assembling A-bodies (Audas et al., 2012a; Jacob, 2013; Audas et al., 2016; Wang et al., 2018) (**Figure 3A**). The various rIGS ncRNA appear to be products of RNA polymerase I (Mayer, 2006; Audas et al., 2012a), except for the antisense PAPAS, which is transcribed by RNA polymerase II (Zhao, 2016).

**Figure 4:**
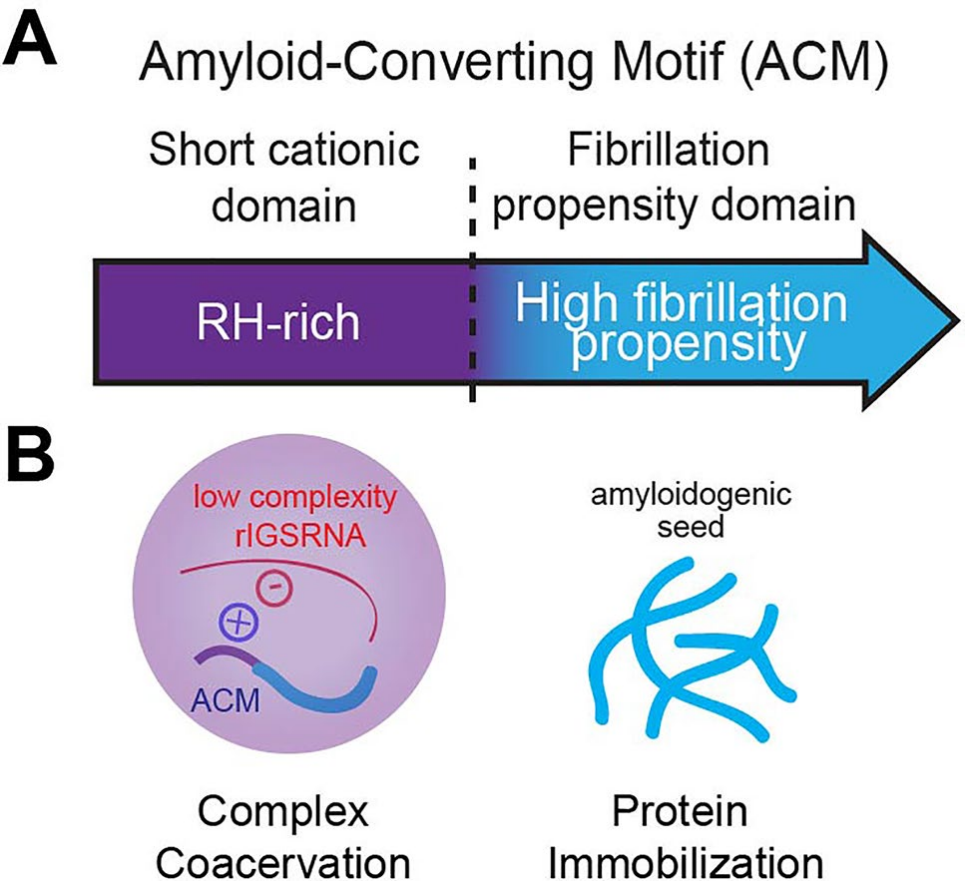
The amyloid-converting motif (ACM). **(A)** The ACM is necessary and sufficient to target and immobilize proteins in amyloid bodies (A-bodies). It consists of a R/H-rich short cationic domain flanking a high fibrillation propensity domain (determined by Rosetta energy of less than −23 kcal/mol). **(B)** We propose that it is its bipartite nature that allows the ACM to traverse phase boundaries. Complex coacervation of short cationic domains and low-complexity rIGSRNA (liquid–liquid phase separation) concentrates fibrillation propensity domains to activate a liquid-to-solid phase transition to form A-bodies.

The appearance of nucleolar foci under stress coincides with the induction of rIGS_28_RNA in acidosis, and rIGS_16_RNA and rIGS_22_RNA in heat shock (**Figure 3**). Recent work suggests that low-complexity dinucleotide repetitive sequences operate as the architectural determinants of rIGSRNA to recruit proteins to A-bodies (Wang et al., 2018) (**Figure 3B**). Live cell imaging and *in vitro* assays indicated that positively charged R/H-rich peptide domains of the ACM (**Figure 4**) co-assemble more efficiently with the negatively charged low-complexity sequences of rIGSRNA than with flanking high-complexity RNA sequences to form the inducible nucleolar foci (Wang et al., 2018). The rIGSRNA/ACM interactions *in vitro* and *in vivo* are particularly sensitive to even the slightest increase in salt concentration. This contrasts other phase-separated compartments of the nucleolus, which are disrupted at considerably higher salt concentrations. Thus, the interactions between low-complexity RNA and the R/H-rich region of the ACM characteristic of step 1 in A-body biogenesis (**Figure 2**) appear to be electrostatic in nature and are likely driven by complex coacervation, a charge-based form of liquid–liquid phase separation that has been observed in other cellular settings *in vivo* (Altmeyer et al., 2015; Pak et al., 2016).

### Amyloidogenic Properties of the ACM Trigger Protein Immobilization

In principle, A-bodies and Balbiani bodies should be composed of proteins that possess fibrillation propensity, i.e., increased likelihood of forming fibrils. Bioinformatic analysis of the consensus ACM revealed the hydrophobic LXV motifs make up a region of high fibrillation propensity, as predicted by a Rosetta energy score of less than −23 kcal/mol (Goldschmidt, 2010) (**Figure 4A**). As a whole, the ACM bears a striking resemblance to that of the prototypical pathological β-amyloid, historically associated with Alzheimer’s disease, in which a R/H-rich sequence is in close proximity to a region of high fibrillation propensity referred to in literature as the P3 fragment (**Figure 4A**) (Dulin et al., 2008). The ACM exhibits classic amyloidogenic properties previously observed for β-amyloid, namely, the cross-β X-ray diffraction pattern *in vivo* and the ability to form fibrils *in vitro* (Audas et al., 2016).

It has been proposed that the function of the initial liquid state is to locally concentrate proteins with fibrillation propensity that would otherwise be at levels below the critical threshold in the cell (Knowles et al., 2014). Concentration-dependent activation of protein fibrillation has been well-documented *in vitro* (Eisenberg and Jucker, 2012; Knowles et al., 2014). Indeed, markers of early or nascent A-bodies include ANS and A11 staining (Wang et al., 2018) that are typically used *in vitro* to indicate accumulating pre-amyloidogenic structures (Booth, 1997; Kayed et al., 2007; Hawe et al., 2008; Tang et al., 2015). In addition, photobleaching analyses revealed that the core of nucleolar foci contain immobile proteins. Nascent A-bodies expand by self-assembly, during which soluble proteins are added directly and autonomously to the growing amyloid structure (**Figure 2**) (Wang et al., 2018).

### The ACM Traverses Across Phase Boundaries to Confer A-Body Identity

As described above, low-complexity RNA sequences are important in conferring A-bodies their unique identity (Wang et al., 2018), adding to the list of architectural determinants, which includes sequence-specific RNA (Yamazaki et al., 2018), mRNA secondary structure (Jain and Vale, 2017; Langdon, 2018), and short unstructured RNA *in vitro* (Nott et al., 2016). The low-complexity rIGSRNA-mediated foci themselves appear immiscible from the three canonical compartments of the nucleolus (Feric et al., 2016; Wang et al., 2018), suggesting they exhibit distinct properties that may exclude mixing. Ultimately, it is the bipartite nature of the ACM that confers A-body identity and differentiates the ACM from other motifs. One possibility is that the R/H-rich “short cationic domain” mediates complex coacervation while the “fibrillation propensity domain” initiates amyloidogenesis to immobilize proteins in A-bodies (Audas et al., 2016; Wang et al., 2018) (**Figure 4B**). In this model, proteins without a region of high fibrillation propensity are unlikely to be incorporated into A-bodies. In other words, while many proteins containing positively charged domains can form transient rIGSRNA-dependent nucleolar foci, only the ones that harbor high fibrillation propensity sequences will be found immobilized in A-bodies. The balance of positive to negative charge ratio, presence of polar and/or aromatic residues, and modifications (Aumiller and Keating, 2016; Monahan et a.l, 2017) will affect how ACM engage in intermolecular interactions with other proteins and low-complexity RNA. Therefore, the ACM is versatile as it can physiologically transition proteins across phase boundaries.

## Physiological Amyloids Promote Cell Dormancy

Endogenous A-bodies have been found in primary cultures and cell lines exposed to various stimuli, the cores of human tumors, and subsets of cells in normal human and mouse tissues (Audas et al., 2016). So, while A-bodies share structural features with pathological amyloids, their ubiquitous and reversible nature is indicative of a physiological function. Pathway enrichment analysis determined that many of the proteins sequestered in A-bodies are involved in cell cycle regulation and DNA synthesis, such as CDK1, POLD1, and DNMT1 (Audas et al., 2016). By temporarily detaining key factors from their sites of activity into A-bodies, major molecular networks are disrupted, suppressing metabolic activity (Mekhail, 2006) and arresting proliferation/DNA synthesis (Audas et al., 2016). This is different from the quiescent state (G0) in the cell cycle where cells arrest proliferation but remain metabolically active. Therefore, the biological role of A-body formation is to promote cellular dormancy as an adaptive response to environmental stressors. These findings are consistent with reports that show Balbiani bodies (Boke et al., 2016) and fluid-to-solid transitions in yeast (Munder et al, 2016) promote dormancy, reinforcing the concept that cells utilize different states of matter to perform various biological functions (**Table 2**). The material properties of “solid-like” systems—non-dynamic, amyloid-like—result in loss of metabolic activity and promote cellular dormancy, while those of ”liquid-like” systems—dynamic, fluid-like—concentrate and facilitate biochemical reactions.

## A-Bodies as Targets for Therapeutic Discovery

### Implications for Pathological Amyloids

A-bodies that are physiologically produced remain confined within the nucleus and are considered non-toxic amyloids (Wang et al., 2017). How the nuclear environment alters the interaction properties of aggregation-prone proteins to prevent toxicity (Woerner et al., 2016; Maharana, 2018) compared to the cytoplasm is unclear. These results imply the cell tightly regulates the induction and degradation of low-complexity rIGSRNA within the nucleolus in response to stimuli. It is interesting that the pathological β-amyloid involved in Alzheimer’s disease is an ACM and undergoes immobilization in A-bodies. Mutations that decrease the fibrillation potential of β-amyloid prevent its immobilization in A-bodies, providing a link between β-amyloid fibrillation and A-body biogenesis. It is tempting to speculate that disruptions of A-body biogenesis, for example by cell death, may provide the initial seeds for pathological amyloidogenesis. Whether aberrant production or localization of low-complexity RNA or exposure of A-bodies to the extracellular environment is involved in amyloid pathologies such as Alzheimer’s and Huntington’s diseases will be the subject of future studies.

### Implications for Tumor Cell Dormancy and Metastasis

Given the evidence that A-bodies are observed in the cores of tumors and promote cell dormancy (Audas et al., 2016), perhaps there is a link between A-body formation and tumor cell dormancy. This could allow cancer cells to adapt to the harsh hypoxic/acidotic conditions of the tumor microenvironment. Such a connection has pertinent implications for chemotherapeutic resistance and metastasis (Li, 2014; Li, 2015). With major metabolic pathways shut down and key drug targets stored away in A-bodies, a small population of dormant tumor cells may be resistant to treatment, survive for a prolonged period of time, and contribute to disease recurrence or metastasis upon their metabolic reactivation. Preventing cancer cells from forming A-bodies and going dormant or, more realistically, preventing cancer cells from reactivating may become a viable treatment option.

An interesting avenue of research that may offer a more effective cancer treatment strategy involves manipulating the proteins that evade capture into A-bodies. Interestingly, proteins that remain active to sustain basal metabolism and viability under stress tend to be devoid of fibrillation propensity domains and evade capture into A-bodies. These proteins would be more susceptible to manipulation and serve as better chemotherapeutic targets than proteins captured in A-bodies.

## Discussion

Twenty years after its discovery, the study of nucleolar sequestration has led to important conceptual and mechanistical advances in our understanding of the role of membraneless bodies and how they are constructed. The ability of cells to reversibly cycle proteins from a mobile to immobile state in A-bodies represents an effective posttranslational mechanism to regulate molecular networks. In this review, we propose a stepwise working model of A-body biogenesis that highlights this process as a precisely choreographed multistep routine rather than a random aggregation of misfolded proteins. This also makes A-bodies unique from the liquid compartments that populate the cell. A-bodies are characterized by electron-dense fibers composed of an array of immobilized proteins that stain positive with various amyloidogenic dyes. Liquid condensates do not display amyloidogenic features and generally contain mobile proteins. Obviously, this raises the key question of why liquid condensates typically do not mature into bodies with amyloidogenic properties. One possibility is that the clusters of low-complexity rIGSRNA are able to recruit sufficient quantity of proteins by simple electrostatic interactions, thereby reaching the critical concentration threshold to trigger amyloidogenesis (Knowles et al., 2014). This model is supported by the observation of electron-dense material early on in A-body biogenesis. The bipartite nature of the ACM, which can both electrostatically interact with low-complexity rIGSRNA and contains a high fibrillation propensity domain necessary for immobilization, may also explain the maturation of A-bodies. Perhaps the architectural determinants of RNA that seed various liquid bodies are too restrictive to certain binding partners to reach a concentration threshold required for amyloidogenesis. Additionally, there is evidence that suggests high nuclear RNA concentration acts as a buffer to prevent condensates from becoming amyloidogenic (Maharana et al., 2018). Indeed, mutated proteins with increased propensity to form amyloids are preferentially formed in the cytoplasm, which has low RNA concentration. While this appears to be the case for cytoplasmic stress granules enriched in amyloidogenic mutant proteins, this likely does not explain A-body maturation as the nucleolus is highly enriched in rRNA. How A-bodies progress from liquid-like condensates to solid-like condensates is a major question in the field of condensate biology, which we are actively pursuing. We are also keen to explore other stimuli, particularly those that alter nucleolar architecture, that may promote the accumulation of rIGSRNA and the formation of A-bodies, including viral infection (Wang, 2010), inflammation, and diurnal cycles of metabolism. Diurnal oscillations in nucleolar size and abundance of nucleolar RNAs have been seen in mammalian systems (Sinturel et al., 2017; Aitken and Semple, 2017), as well as an emerging connection between circadian disturbances and Alzheimer’s disease (Musiek, 2015). Despite most proteins having inherent fibrillation propensity, the dominant view remains that amyloids are inherently toxic, rather than a physiological fold exploited by cells to regulate various peptide functions. The challenge will be to decipher fundamental differences between A-bodies and other physiological or pathological membraneless compartments that could inform our understanding on how to prevent, detect, and treat amyloid-based diseases.

## Author Contributions

MW, MB, PT, and SL designed the manuscript, discussed the conceptual implications, wrote the paper, and made the figures.

## Funding

This work was supported by grants from the National Institute of General Medical Sciences (R01GM115342) (SL), the National Cancer Institute (R01CA200676) of the NIH, and the Sylvester Comprehensive Cancer Center (SL).

## Conflict of Interest

The authors declare that the research was conducted in the absence of any commercial or financial relationships that could be construed as a potential conflict of interest.
